# Associations of dietary intake and nutrition knowledge with body composition and performance in table tennis athletes: a cross-sectional study

**DOI:** 10.3389/fnut.2026.1858347

**Published:** 2026-07-03

**Authors:** Shui-Jian Deng, Jiang Yu, Meng-Yuan Shu, Guoqi Li, Yunrui Liu, Lintao Wang, Mingyi Ma, Guzalinur Yushan, Yiqian Li, Yangqin Tan, Yulu Su, Tao Ye, Chang He, Xiu-Chang Zhang, Jian Liang

**Affiliations:** 1 School of Sports Science, Hengyang Normal University, Hengyang, China; 2 Department of Laboratory Medicine, Xiangnan Hospital Affiliated to Hunan Normal University/922th Hospital of Joint Logistics Support Force, PLA, Hengyang, China; 3 School of Physical Education, Kashi University, Kashi, China; 4 Experimental Teaching Demonstration Center of Food Safety and Nutrition, Xinjiang Institute of Technology, Aksu, China; 5 Aksu Institute of Apple, Xinjiang Institute of Technology, Aksu, China; 6 Department of Biology and Medicine, Hengyang Normal University, Hengyang, China; 7 Department of Biological Science, Hengyang Normal University, Hengyang, China; 8 Department of Biology, Soonchunhyang University, Asan, Republic of Korea

**Keywords:** bioelectrical impedance analysis (BIA), body composition, dietary intake, nutrition knowledge, table tennis athlete

## Abstract

**Background:**

Table tennis is a high-intensity intermittent sport requiring rapid movement, cognitive processing, and repeated recovery. Adequate nutrition may therefore be important for supporting athletes’ health and performance. However, limited research has simultaneously examined dietary intake, nutrition knowledge, body composition, and physical performance in table tennis athletes.

**Objective:**

This study aimed to characterize dietary intake and nutrition knowledge among trained table tennis athletes and to explore their associations with body composition and selected physical performance indicators.

**Methods:**

This cross-sectional study included 42 trained table tennis athletes. Dietary intake was assessed using three non-consecutive 24-h recalls, and nutrition knowledge was evaluated using a structured questionnaire. Body composition was measured using bioelectrical impedance analysis. Physical performance was assessed using the standing long jump, reaction time, and shuttle run tests. Correlation analysis, group comparisons, multiple linear regression, and principal component analysis (PCA) were conducted.

**Results:**

Macronutrient distribution was generally within recommended ranges, whereas reported energy intake, dietary fiber, vitamin C, calcium, iron, folate, and fruit and vegetable intake were below recommended levels. Nutrition knowledge was average, with relatively lower scores in the sports nutrition and supplement-use domains. No significant differences were observed between the high- and low-nutrition knowledge groups in standing long jump, reaction time, shuttle run performance, fat-free mass, muscle mass, or total body water. Regression analyses identified limited independent associations between dietary or body composition variables and performance outcomes. No significant predictors of standing long jump or reaction time were identified. Age was associated with shuttle run time (*p* = 0.048), but the model had limited explanatory power (adjusted R^2^ = 0.068). The first two principal components explained 64.8% of the total variance, and the PCA showed substantial overlap between the high- and low-nutrition knowledge groups.

**Conclusion:**

Trained table tennis athletes demonstrated suboptimal dietary quality despite generally adequate macronutrient distribution. Within this sample, nutrition knowledge was not significantly associated with body composition or selected physical performance indicators. Given the small sample size and cross-sectional design, these findings should be regarded as exploratory. Larger longitudinal studies incorporating sport-specific performance measures and objective assessments of energy expenditure are warranted.

## 1 Introduction

Table tennis is a high-intensity intermittent sport characterized by rapid, explosive movements combined with short recovery periods (1, 2). Although individual rallies are brief, matches often involve repeated bouts of high-speed action that require both anaerobic and aerobic energy systems (3). Such physiological demands underscore the importance of appropriate nutritional strategies to support performance, recovery, and overall health (4, 5).

Optimal dietary intake is a key determinant of athletic performance (6). Adequate energy availability, macronutrient balance, and micronutrient sufficiency are essential for maintaining muscle function, cognitive performance, and fatigue resistance (7, 8). However, previous studies have indicated that athletes, particularly those in racket sports, frequently fail to meet recommended nutritional guidelines (9). For example, a study of competitive table tennis players reported widespread inadequacies in dietary intake, particularly in micronutrient intake, alongside generally poor dietary habits (10).

In addition to dietary intake, nutrition knowledge has been identified as an important factor influencing athletes’ eating behaviors (11, 12). Athletes with higher levels of nutrition knowledge are more likely to make appropriate dietary choices, which may positively affect their body composition and performance (13, 14). Nevertheless, evidence suggests that many athletes have only average or insufficient knowledge of nutrition, and the relationship between knowledge and actual dietary behavior remains inconsistent across studies. Body composition is another critical component influencing performance in table tennis. Lower body fat and higher lean mass have been associated with improved speed, agility, and power output, all of which are essential for successful performance in this sport (15, 16). Since dietary intake directly affects body composition, and nutrition knowledge may influence dietary habits, these variables are likely interconnected (17). However, despite their potential importance, studies examining these relationships simultaneously remain limited. Recent evidence from university athletes also indicates that sports nutrition knowledge and food habits are shaped by social and demographic factors, supporting the need to interpret knowledge–behavior relationships within athletes’ broader food environments (18).

Although previous studies have provided important insights into dietary intake, nutrition knowledge, body composition, and performance as separate domains in table tennis and other athletic populations (19, 20), these factors are often examined independently. The present study evaluates dietary intake, nutrition knowledge, body composition, and physical performance indicators within a single analytical framework, while also considering sex as a potential biological and behavioral source of variation. This approach allows for a more comprehensive assessment of the relationships among nutrition-related variables, body composition, and general physical-performance indicators in trained table tennis athletes.

Therefore, the present study aimed to investigate dietary intake and nutritional knowledge among table tennis athletes and to examine their associations with body composition and physical performance in a cross-sectional design. By simultaneously evaluating these interrelated domains, this study seeks to provide a more comprehensive characterization of the nutritional and physiological profiles of table tennis athletes and to offer evidence to inform targeted nutritional strategies for this population.

## 2 Materials and methods

### 2.1 Study design

This study employed a cross-sectional design to investigate the relationships among dietary intake, nutrition knowledge, body composition, and physical performance in table tennis athletes. All measurements were conducted within a single testing session under standardized conditions. Participants completed a nutrition knowledge questionnaire, dietary assessment, body composition analysis, and physical performance tests. This study was approved by the Institutional Ethics Committee of Hengyang Normal University (approval no. 2026LIIW009). The study was conducted in accordance with relevant ethical guidelines and regulations. All participants provided written informed consent before participation.

### 2.2 Participants

A total of 55 table tennis athletes from Hengyang City, China, were initially assessed for eligibility through online advertisements and public announcements. Participants were required to meet the following inclusion criteria: (1) participation in at least provincial-level competitions, (2) a minimum of 5 years of training experience, and (3) a weekly training duration of at least 10 h.

Based on these criteria, 11 athletes were excluded, including those without provincial-level competition experience (*n =* 5), those with <5 years of training experience (*n =* 3), and those younger than 18 years of age (*n =* 3). Consequently, 44 eligible athletes were enrolled in the study.

**Figure 1:**
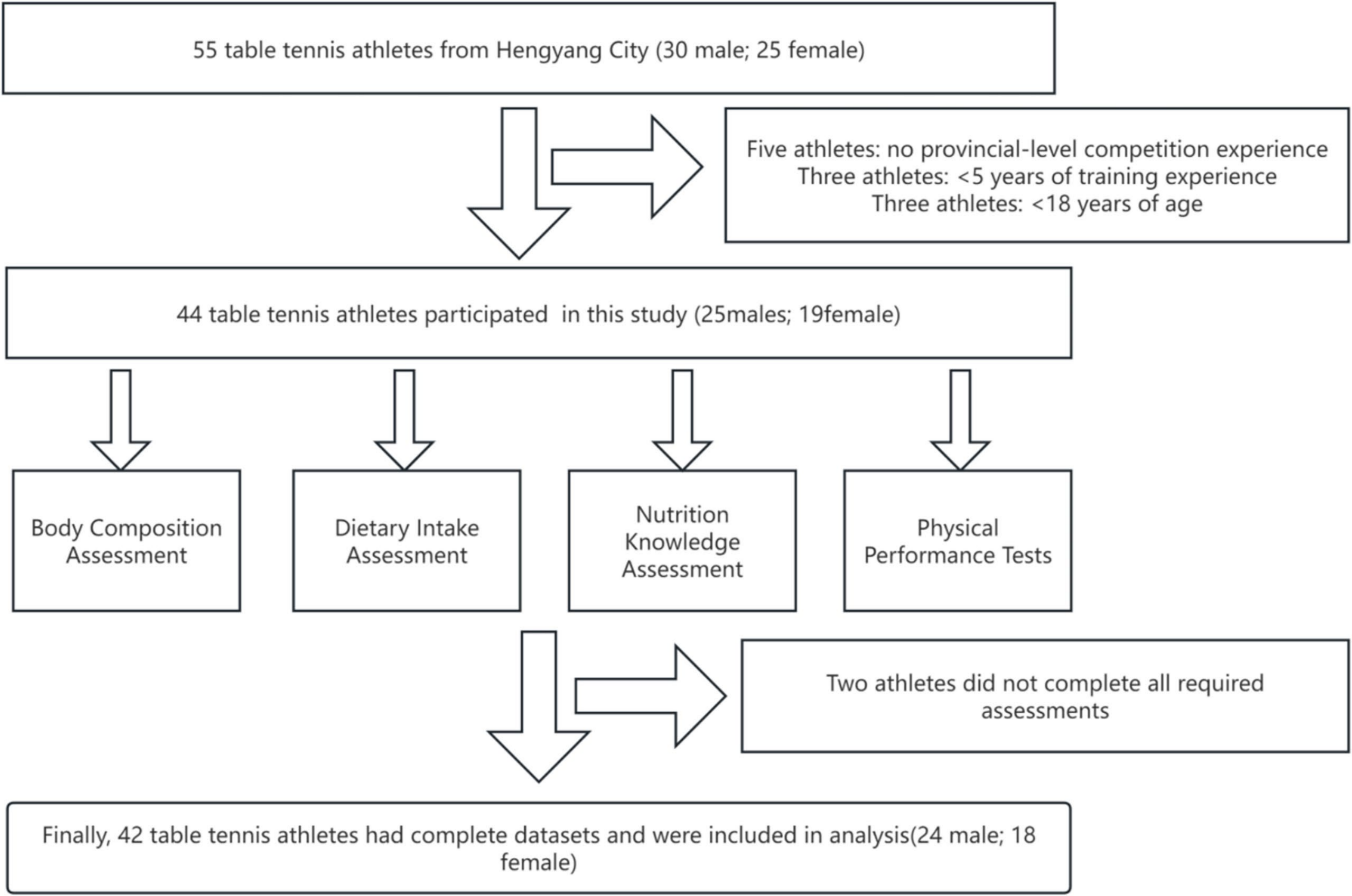
Flow diagram of participant recruitment and data inclusion.

During the study, two participants did not complete all required assessments and were therefore excluded from the final analysis. Ultimately, data from 42 athletes were included in the final analysis. The participant selection process is illustrated in Figure 1, and the general characteristics of the participants are presented in Table 1.

**Table 1:** General characteristics of the participants.

General characteristics	Table tennis athletes
Sex	Male (*n =* 24), Female (*n =* 18)
Age (year)	18–24 (21.0 ± 1.5)
Weight (kg)	38.0–96.8 (66.9 ± 13.7)
Height (cm)	148–180 (170.3 ± 8.1)

### 2.3 Dietary intake assessment

Dietary intake was assessed using Intake24, an online, self-administered 24-h dietary recall system.^1^ Intake24 is a web-based tool that uses the multiple-pass method to facilitate detailed, accurate recall of all foods and beverages consumed the previous day. To better reflect habitual dietary intake and reduce day-to-day variability, dietary data were collected over three non-consecutive days, including two weekdays and one weekend day. Participants were instructed to report all meals, snacks, and beverages consumed every 24 h. The system provides a structured, step-by-step interface with prompts, portion-size images, and food-search functions to improve recall accuracy and minimize reporting bias. Nutrient intake was automatically calculated using standardized food composition databases embedded within the system. The average intake across the 3 days was used for analysis. The following variables were extracted: total energy intake (kcal), macronutrients (fat, saturated fat, and sugar), dietary fiber (g), and selected micronutrients (vitamin C, calcium, iron, and folate). Intake24 has been widely used in dietary assessment research and has demonstrated acceptable validity and reliability when compared with interviewer-administered 24-h recalls and objective measures of energy expenditure (21).

### 2.4 Nutrition knowledge assessment

Nutrition knowledge was assessed using a structured sports nutrition questionnaire consisting of six domains: weight management, macronutrients, micronutrients, sports nutrition, supplementation, and alcohol. The questionnaire was developed to evaluate both general and sport-specific nutrition knowledge relevant to athletes and has demonstrated acceptable validity and reliability in athletic populations (22, 23). Each item was scored according to the published scoring guidelines, and domain-specific and total scores were calculated, with higher scores indicating greater nutrition knowledge.

Total scores were converted into percentages of the maximum possible score and interpreted according to the classification commonly applied in NSKQ/A-NSKQ research: <50% was classified as poor, 50–65% as average, 66–75% as good, and >75% as excellent nutrition knowledge (23). These classification thresholds have been widely adopted in studies involving athletes from different sports and competitive levels. Participants completed the questionnaire independently under standardized conditions. For exploratory group comparisons, participants were additionally categorized into relatively high- and low-scoring groups based on the sample median. This median split reflected relative nutrition knowledge within the present sample and was not used to define absolute knowledge levels.

The NSKQ and A-NSKQ have subsequently been translated into several languages, including Chinese; however, no published study has specifically validated the questionnaire among Chinese competitive table tennis athletes. Therefore, the present study primarily used the questionnaire to compare relative differences in nutrition knowledge within this athletic population, and the absolute score classifications should be interpreted with caution (24). Nutrition knowledge is an important component of sports nutrition research because greater nutrition knowledge may contribute to healthier dietary behaviors, improved dietary quality, and more effective nutritional practices that support athletic performance and recovery (25).

### 2.5 Body composition assessment

Body composition was assessed using a bioelectrical impedance analyzer (BIA) (Jinghai S-one+5 s, Jinhai Inc., Beijing, China) under standardized testing conditions. Participants were instructed to refrain from strenuous exercise, alcohol consumption, and food intake for at least 8–12 h before assessment. All measurements were performed in the morning following an overnight fast to minimize the influence of hydration status, recent dietary intake, glycogen-associated fluid shifts, and circadian variation on impedance measurements. Participants were also instructed to maintain their habitual dietary and training routines throughout the study period and to avoid vigorous exercise for 24 h before each assessment. During testing, participants stood barefoot on the analyzer and maintained a standardized posture according to the manufacturer’s instructions. Although bioelectrical impedance analysis may be influenced by hydration status, glycogen fluctuations, and exercise-induced changes in body fluid distribution, standardized pre-assessment procedures were implemented to reduce measurement variability and improve reliability. The following variables were included in the analysis: muscle mass (kg), fat-free mass (kg), and total body water (kg). These parameters are widely used indicators of body composition and are closely associated with physical performance in athletes.

### 2.6 Physical performance tests

Physical performance was assessed using standardized field-based tests, including the 5–10–5 shuttle run (Pro-Agility Test), standing long jump, and reaction time test, to evaluate agility, lower-limb explosive power, and neuromuscular response ability, respectively. All tests were conducted indoors under standardized conditions. Before testing, participants completed a 10–15-min standardized warm-up, including dynamic stretching and familiarization trials. For the 5–10–5 shuttle run, three parallel lines were marked 5 m apart, with the middle line serving as the starting point. Participants sprinted 5 m to one side, then 10 m to the opposite side, and finally returned 5 m to the starting line. The time was recorded using a stopwatch (accuracy 0.01 s), and the best of three trials was used for analysis, with 2–3 min rest intervals between trials. The standing long jump was used to assess lower-limb explosive power. Participants performed a maximal horizontal jump from a standing position with a two-foot take-off. Three trials were conducted with 1–2 min rest intervals, and the best performance was recorded. Reaction time was assessed using a standardized testing device (GMCS-FYS3, Jianmin, China). Participants were instructed to respond as quickly as possible to a visual stimulus. Multiple trials were performed, and the average reaction time was calculated for analysis. All participants were instructed to perform at maximal effort during each test, and sufficient rest was provided between trials to minimize fatigue. These physical capacities were selected because table tennis involves repeated multidirectional movements, rapid lower-limb force production, and fast responses to visual stimuli. Although these tests do not directly assess table tennis-specific technical or tactical performance, they provide practical, reproducible measures of general physical capacities that may support movement efficiency and performance in table tennis training and competition.

### 2.7 Statistical analysis

No *a priori* power analysis was performed because recruitment was constrained by the limited availability of eligible competitive table tennis athletes who met the competition-level, training-experience, and assessment-completion criteria. Therefore, the sample size was determined by participant availability and the availability of complete-case data. Given the final sample of 42 athletes, all analyses were considered exploratory rather than confirmatory. The limited statistical power may have prevented the detection of small effects; therefore, non-significant results should not be interpreted as evidence of no association.

All statistical analyses were performed using R (version 4.0) and GraphPad Prism (version 10.0). Data distributions were assessed using the Shapiro–Wilk test. Normally distributed continuous variables are presented as mean ± standard deviation (SD). Non-normally distributed variables were analyzed using appropriate non-parametric methods.

Sex-stratified descriptive analyses were conducted, and male–female comparisons were performed using Welch’s *t*-tests. *p*-values, 95% confidence intervals (CIs) for mean differences, and Cohen’s d effect sizes were reported. For exploratory comparisons based on nutrition knowledge, participants were categorized into relatively high- and low-scoring groups using the sample median as the cutoff. This categorization reflected relative levels of nutritional knowledge within the present sample and did not represent absolute knowledge levels. Differences in body composition and physical performance between these groups were evaluated using Welch’s *t*-tests or Mann–Whitney U tests, as appropriate.

Associations among nutrition knowledge, dietary intake, body composition, and physical performance variables were examined using Spearman’s rank correlation coefficients. Principal component analysis (PCA) was performed as an exploratory dimensionality-reduction technique to summarize multivariate patterns among dietary intake and body composition variables. All variables were standardized using z-scores before the PCA.

Data suitability for PCA was assessed using the Kaiser-Meyer-Olkin (KMO) measure of sampling adequacy and Bartlett’s test of sphericity. An overall KMO value >0.60 and a statistically significant Bartlett’s test were considered to indicate general suitability for PCA. Individual measures of sampling adequacy were also examined, with values below 0.50 indicating potentially inadequate representation within the component structure. Given the relatively small participant-to-variable ratio, the PCA was considered exploratory. Components were interpreted according to explained variance, loading patterns, and biological plausibility.

Multiple linear regression analyses were performed using ordinary least squares to examine the independent associations of selected dietary, body composition, and demographic variables with standing long-jump, reaction time, and shuttle run outcomes. Predictors were selected based on theoretical relevance and observed correlation patterns, and were entered simultaneously into each model.

Multicollinearity was assessed using variance inflation factors (VIFs), with values >5 indicating potentially problematic multicollinearity. Regression assumptions, including linearity, homoscedasticity, independence, and residual normality, were evaluated using residual diagnostics. Model performance was assessed using R^2^ and adjusted R^2^. Unstandardized coefficients (B), standard errors (SEs), 95% CIs, *p*-values, and VIF values were reported.

To address multiplicity, *p*-values were adjusted using the Benjamini-Hochberg false discovery rate procedure separately within four prespecified families of analyses: sex-stratified comparisons, nutrition-knowledge group comparisons, regression coefficients, and pairwise Spearman correlations. Statistical significance after correction was defined as a Benjamini-Hochberg-adjusted *p*-value (*q*-value) < 0.05. Raw *p*-values are provided for transparency, but interpretations were based primarily on the adjusted results.

## 3 Results

### 3.1 Results of daily dietary intake

The daily dietary intake of table tennis athletes is presented in Table 2. Total energy intake exhibited considerable inter-individual variability, ranging from 242.7 to 2721.9 kcal/day, with a mean intake of 1837.6 ± 507.2 kcal/day. Regarding macronutrient composition, mean total fat intake accounted for 24.0 ± 14.5% of total energy intake, which was within the recommended range of 20–35%. Likewise, mean saturated fat intake (7.9 ± 5.5% of total energy) and free sugar intake (5.0 ± 10.4% of total energy) were below the recommended upper limit of 10%, although substantial variability was observed among participants.

**Table 2:** Daily dietary intake of table tennis athletes compared with recommended values.

Variable	Range	Mean ± SD	Recommended value	Achievement (%)	Interpretation	References
Total energy intake (kcal)	242.7–2721.9	1837.6 ± 507.2	/	Not calculated	No single reference target	/
Total fat intake (%)	1.9–51.4	24.0 ± 14.5	20–35% of total energy	Within range	Adequate mean proportion	(41)
Saturated fat intake (%)	0.5–21	7.9 ± 5.5	<10% of total energy	79% of the upper limit	Within the recommended limit	(41)
Sugar intake (%)	0–57.7	5.0 ± 10.4	<10% of total energy	50% of the upper limit	Within the recommended limit	(42)
Dietary fiber (g)	0.7–30.0	8.0 ± 6.1	≥25–38 g/day	21–32%	Insufficient	(42)
Vitamin C (mg)	0–130.1	24.1 ± 33.0	75–90 mg/day	27–32%	Insufficient	(43)
Calcium (mg)	29.4–1098.2	331.5 ± 261.7	800–1,000 mg/day	33–41%	Insufficient	(43)
Iron (mg)	0–13.6	4.3 ± 3.3	8–18 mg/day	24–54%	Insufficient	(43)
Folate (μg)	6.7–277	84.4 ± 68.6	400 μg/day	21%	Insufficient	(43)
Red meat intake (g)	0–208	35.1 ± 47.6	Moderate intake	Not calculated	Qualitative recommendation	(42)
Fruit and vegetable intake	0–5.6	1.4 ± 1.3	≥5 servings/day	28%	Insufficient	(42)

Achievement percentages are descriptive ratios of the sample mean to the stated reference value or range. They are not inferential tests and should be interpreted with caution, as some recommendations vary by sex and individual energy requirements.

In contrast, the intake of several essential micronutrients and dietary fiber was markedly inadequate. Mean dietary fiber intake was only 8.0 ± 6.1 g/day, considerably lower than the recommended 25–38 g/day. Similarly, average intakes of vitamin C (24.1 ± 33.0 mg/day), calcium (331.5 ± 261.7 mg/day), iron (4.3 ± 3.3 mg/day), and folate (84.4 ± 68.6 μg/day) were all below their respective recommended dietary intakes. Food group analysis further revealed insufficient consumption of fruits and vegetables, with participants reporting an average intake of only 1.4 ± 1.3 servings/day, substantially lower than the recommended minimum of five servings per day. Red meat consumption showed substantial variation, ranging from 0 to 208 g/day. To further evaluate dietary adequacy, nutrient intake was expressed as a percentage of established dietary recommendations. Dietary fiber intake was only 32.0% of the minimum recommended intake (25 g/day), while vitamin C, calcium, iron, and folate were 32.1, 41.4, 53.8, and 21.1% of their respective recommended levels. Fruit and vegetable consumption accounted for only 28.0% of the recommended 5 servings per day. Collectively, these findings indicate that although the overall macronutrient distribution generally conformed to current dietary guidelines, most athletes failed to meet recommendations for dietary fiber, key micronutrients, and fruit and vegetable intake, highlighting potential nutritional inadequacies within this population.

### 3.2 Results of nutrition knowledge

The nutrition knowledge scores of table tennis athletes are presented in Table 3. The total nutrition knowledge score ranged from 6.0 to 75.0, with a mean value of 39.5 ± 11.3, indicating an average level of nutrition knowledge among participants. Among the six domains, the highest scores were observed in macronutrients (14.2 ± 4.9) and micronutrients (6.5 ± 2.6), suggesting a relatively better understanding of basic nutritional components. In contrast, lower scores were found in sports nutrition (4.1 ± 2.4) and supplement use (4.4 ± 2.6), indicating limited knowledge in more specialized areas of sports nutrition. Scores for weight management (5.8 ± 2.0) and alcohol (4.5 ± 1.8) were average, reflecting a basic awareness of general health-related nutrition topics.

**Table 3:** Nutrition knowledge scores of table tennis athletes.

Domain	Range	Mean ± SD
Weight management	2.0–12.0	5.8 ± 2.0
Macronutrients	0.0–27.0	14.2 ± 4.9
Micronutrients	0.0–12.0	6.5 ± 2.6
Sports nutrition	0.0–10.0	4.1 ± 2.4
Supplement use	0.0–10	4.4 ± 2.6
Alcohol	0.0–8.0	4.5 ± 1.8
Total score	6.0–75.0	39.5 ± 11.3

Maximum possible total score: 79; Mean percentage score: 50.0% Classification: Average.

### 3.3 Body composition

The body composition of table tennis athletes is presented in Table 4. Fat-free mass ranged from 35.0 to 67.9 kg, with a mean value of 52.5 ± 8.0 kg. Muscle mass ranged from 32.7 to 63.9 kg, with a mean of 49.0 ± 7.5 kg. Total body water ranged from 25.1 to 50.3 kg, with a mean value of 37.9 ± 6.2 kg. Overall, the participants demonstrated relatively high levels of fat-free mass and muscle mass, reflecting their well-trained status.

**Table 4:** Body composition of table tennis athletes.

Body composition	Range	Mean ± SD
Fat-free mass	35.0–67.9	52.5 ± 8.0
Muscle mass	32.7–63.9	49.0 ± 7.5
Total body water	25.1–50.3	37.9 ± 6.2

### 3.4 Results of physical performance

The physical performance outcomes of table tennis athletes are presented in Table 5. Standing long jump performance ranged from 1.5 to 2.6 m, with a mean value of 2.0 ± 0.3 m, indicating a relatively good level of lower-limb explosive power among participants. Reaction time ranged from 0.5 to 0.7 s, with a mean value of 0.6 ± 0.1 s, reflecting moderate neuromuscular response ability. Shuttle run performance ranged from 6.3 to 8.7 s, with an average time of 7.1 ± 0.6 s.

**Table 5:** Physical performance outcomes of table tennis athletes.

Physical performance	Range	Mean ± SD
Standing long jump	1.5–2.6	2.0 ± 0.3
Reaction time	0.5–0.7	0.6 ± 0.1
Shuttle run	6.3–8.7	7.1 ± 0.6

### 3.5 Sex-stratified comparisons

Sex-stratified comparisons are presented in Table 6. After Benjamini-Hochberg false discovery rate correction, male athletes had significantly greater fat-free mass, muscle mass, and total body water than female athletes (all raw *p <* 0.001; all *q <* 0.001), with large effect sizes (Cohen’s *d =* 2.65–2.72). The corresponding mean differences were 12.9 kg for fat-free mass (95% CI: 9.85–15.90), 11.9 kg for muscle mass (95% CI: 9.08–14.82), and 10.0 kg for total body water (95% CI: 7.67–12.36). Male athletes also reported higher absolute energy intake than female athletes (1981.2 ± 594.6 vs. 1646.2 ± 272.1 kcal/day; raw *p* = 0.020, q = 0.048; *d =* 0.69). Although dietary fiber intake was higher among males in the unadjusted analysis (9.5 ± 7.2 vs. 6.0 ± 3.5 g/day; raw *p* = 0.043), this difference did not remain significant after FDR correction (q = 0.086; *d =* 0.60). No significant sex differences were identified in energy intake relative to body mass, vitamin C intake, iron intake, or nutrition knowledge scores after correction (all q > 0.05). Male athletes performed significantly better in the standing long jump than female athletes (2.19 ± 0.20 vs. 1.78 ± 0.19 m; raw *p <* 0.001, *q <* 0.001), with a very large effect size (*d =* 2.05). No significant sex differences were observed in reaction time or shuttle-run performance after FDR correction (q = 0.203 and q = 0.825, respectively). Although reaction time showed a moderate effect size favoring males (*d =* −0.50), this difference was not statistically significant.

**Table 6:** Sex-stratified descriptive characteristics, dietary intake, body composition, and performance outcomes.

Variable	Male (*n =* 24)	Female (*n =* 18)	*P*-value	BH-FDR *p*	95% CI of the difference	Effect size (*d*)
Fat-free mass (kg)	58.1 ± 4.6	45.2 ± 4.9	<0.001	0	9.85 to 15.90	2.72
Muscle mass (kg)	54.1 ± 4.4	42.2 ± 4.6	<0.001	0	9.08 to 14.82	2.65
Total body water (kg)	42.2 ± 3.6	32.2 ± 3.8	<0.001	0	7.67 to 12.36	2.71
Energy intake (kcal/day)	1981.2 ± 594.6	1646.2 ± 272.1	0.020	0.048	56.09 to 614.02	0.69
Energy intake (kcal/kg/day)	27.4 ± 8.4	27.8 ± 6.8	0.369	0.4227	−2.61 to 6.89	0.28
Dietary fiber (g/day)	9.5 ± 7.2	6.0 ± 3.5	0.043	0.0861	0.12 to 6.94	0.60
Vitamin C (mg/day)	26.4 ± 37.4	21.0 ± 26.9	0.585	0.6378	−14.59 to 25.52	0.16
Iron (mg/day)	4.8 ± 3.8	3.6 ± 2.4	0.208	0.2922	−0.71 to 3.18	0.37
Nutrition knowledge score	37.8 ± 12.9	41.9 ± 8.5	0.219	0.2922	−10.84 to 2.56	−0.37
Standing long jump (m)	2.19 ± 0.20	1.78 ± 0.19	<0.001	0	0.28 to 0.53	2.05
Reaction time (s)	0.57 ± 0.06	0.60 ± 0.06	0.118	0.2026	−0.07 to 0.01	−0.50
Shuttle run (s)	7.10 ± 0.51	7.15 ± 0.72	0.825	0.8246	−0.45 to 0.36	−0.07

Values are mean ± SD. Sex-stratified comparisons were conducted using Welch’s *t*-tests. Effect size is Cohen’s *d*.

### 3.6 Multivariate and exploratory analyses

#### 3.6.1 Results of correlation analysis

**Figure 2:**
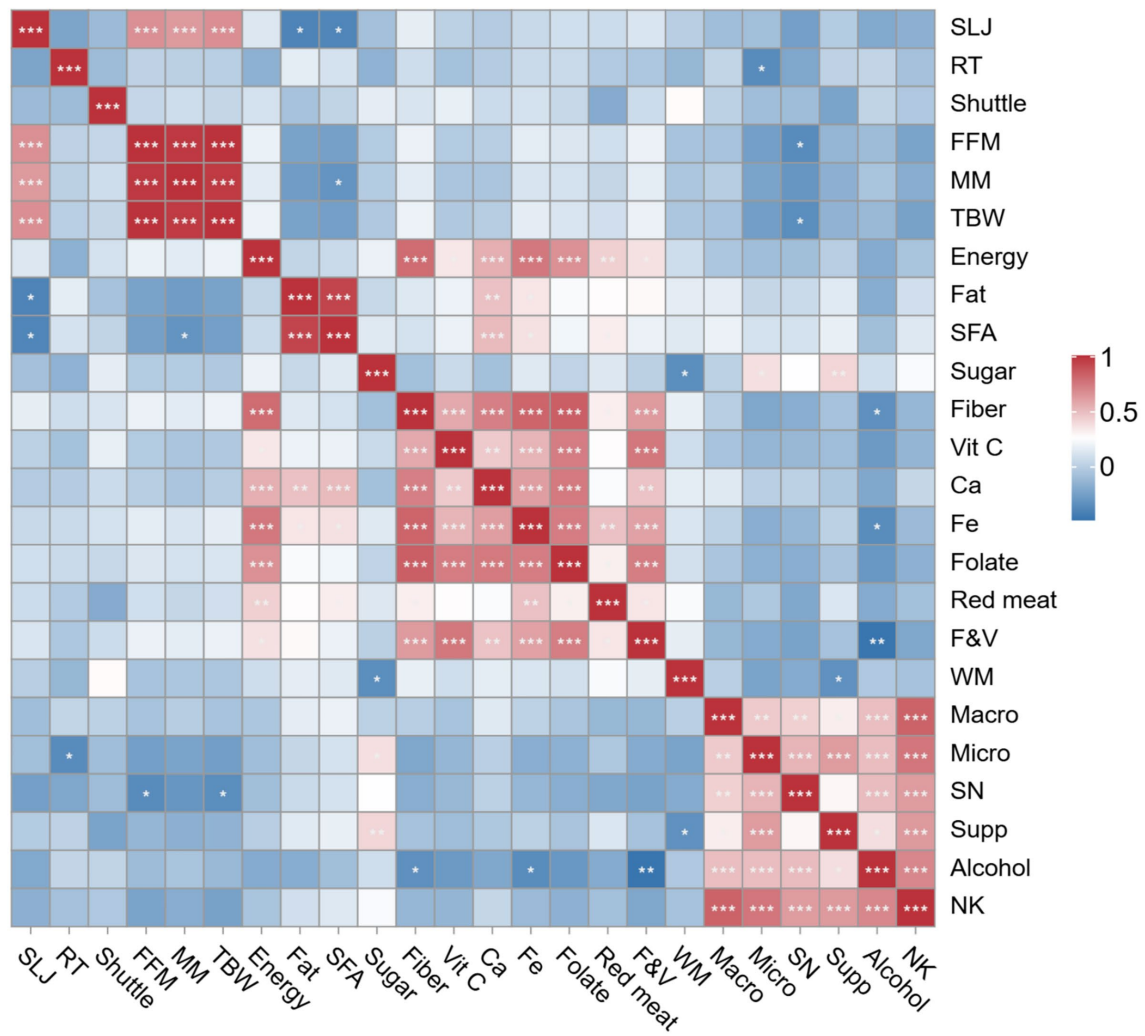
Correlation heatmap showing associations among physical performance, body composition, dietary intake, and nutrition-related variables in table tennis athletes. Heatmap illustrating the pairwise correlations among physical performance variables (SLJ, standing long jump; RT, reaction time; Shuttle, shuttle run), body composition indicators (FFM, fat-free mass; MM, muscle mass; TBW, total body water), dietary intake variables (energy, fat, SFA, sugar, fiber, vitamin C, calcium, iron, folate), food categories [red meat, fruits and vegetables (F&V), white meat (WM)], and nutrition-related scores [macro-, micro-, sports nutrition (SN), supplement use (Supp), alcohol consumption, and nutrition knowledge (NK)]. Color intensity represents the strength and direction of correlations (re*d =* positive, blue = negative). Asterisks indicate statistical significance (*p < 0.05, **p < 0.01, ***p < 0.001).

Correlation analysis demonstrated clear clustering patterns among the measured variables (Figure 2). Body composition indicators, including fat-free mass (FFM), muscle mass (MM), and total body water (TBW), were strongly and positively correlated with each other (all *p <* 0.001) and showed positive associations with standing long jump (SLJ), indicating a link between lean mass and explosive performance. Dietary variables also exhibited structured relationships, with energy intake positively correlated with fat and saturated fatty acids (SFA), and micronutrients (vitamin C, calcium, iron, and folate) strongly intercorrelated, reflecting consistent dietary patterns. Fiber intake was positively associated with fruit and vegetable consumption, whereas red meat showed weaker associations with micronutrient intake. In addition, nutrition knowledge domains (macro, micro, and sports nutrition) were highly interrelated (*p <* 0.001) and moderately associated with supplement use. At the same time, alcohol consumption tended to show weak or negative correlations with nutrition-related variables.

#### 3.6.2 Results of multiple linear regression analysis

The multiple linear regression analyses revealed generally limited independent associations between the selected predictors and physical performance outcomes (Tables 7–9). For the standing long jump, the regression model explained a moderate proportion of variance (R^2^ = 0.535, adjusted R^2^ = 0.470). However, none of the examined variables, including muscle mass, body fat mass, energy intake, sex, and age, were significantly associated with standing long jump performance (all *p >* 0.05). Although sex showed the largest estimated coefficient among the predictors (B = 0.3598, *p* = 0.107), the association did not reach statistical significance. For reaction time, the regression model demonstrated relatively low explanatory power (R^2^ = 0.152, adjusted R^2^ = 0.060). None of the included variables were significantly associated with reaction time (all *p >* 0.05). Vitamin C intake showed a marginal association (B = −0.0005, *p* = 0.088); however, this finding did not remain significant after correction for multiple comparisons (BH-FDR *p* = 0.465) and should therefore be interpreted cautiously as an exploratory trend only. For shuttle run performance, the model explained a relatively small proportion of variance (R^2^ = 0.159, adjusted R^2^ = 0.068). Among the included predictors, age was the only variable significantly associated with shuttle run time (B = −0.1696, *p* = 0.013; BH-FDR *p* = 0.048), indicating that older athletes tended to run faster shuttles. In contrast, body fat mass, muscle mass, and sex were not significant predictors (all *p >* 0.05). Overall, the regression findings suggest that the examined nutritional intake and body composition variables exhibited limited independent predictive value for physical performance outcomes in the present sample. Furthermore, the relatively low explanatory power of the reaction time and shuttle run models suggests that additional factors not included in the current analyses may contribute to variation in performance.

**Table 7:** Multiple linear regression analysis of factors associated with the standing long jump.

Predictor	B	SE	*P*-value	BH-FDR p	95% CI
Intercept	1.4315	0.8108	0.086	/	−0.2134 to 3.0764
MM	0.0043	0.0160	0.789	0.947	−0.0283 to 0.0370
BFM	−0.0006	0.0090	0.948	0.946	−0.0189 to 0.0177
Energy	−0.000046	0.000076	0.543	0.943	−0.000200 to 0.000107
Sex	0.3598	0.2179	0.107	0.465	−0.0822 to 0.8019
Age	0.0100	0.0237	0.674	0.948	−0.0378 to 0.0578
Model fit: R^2^ = 0.535, adjusted R^2^ = 0.470					

B, unstandardized regression coefficient; SE, standard error; CI, confidence interval; MM, muscle mass; BFM, body fat mass. *P*-values were adjusted for multiple testing using the Benjamini–Hochberg false discovery rate (BH-FDR) procedure. No evidence of problematic multicollinearity was observed, with all variance inflation factor (VIF) values below 5 (MM = 3.07, BFM = 1.17, energy intake = 1.30, sex = 4.09, age = 1.22). The final model explained 53.5% of the variance in performance outcomes (R^2^ = 0.535; adjusted R^2^ = 0.470).

**Table 8:** Multiple linear regression analysis of factors associated with Reaction time.

Predictor	B	SE	*P*-value	BH-FDR p	95% CI
Intercept	0.6725	0.1319	<0.001	/	0.4053 to 0.9397
Sugar	−0.0008	0.0010	0.385	0.875	−0.002519 to 0.000994
Vit C	−0.0005	0.0003	0.088	0.465	−0.001028 to 0.000074
Sex	−0.0257	0.0190	0.184	0.598	−0.0642 to 0.0128
Age	−0.0027	0.0063	0.675	0.952	−0.0155 to 0.0102
Model fit: R^2^ = 0.152, adjusted R^2^ = 0.060					

B, unstandardized regression coefficient; SE, standard error; CI, confidence interval; Vit C, vitamin C intake. *p*-values were adjusted for multiple testing using the Benjamini–Hochberg false discovery rate (BH-FDR) procedure. No evidence of problematic multicollinearity was observed, with all variance inflation factor (VIF) values below 5 (sugar = 1.03, vitamin C = 1.01, sex = 1.13, age = 1.13). The final model explained 15.2% of the variance in performance outcomes (R^2^ = 0.152; adjusted R^2^ = 0.060).

**Table 9:** Multiple linear regression analysis of factors associated with the shuttle run.

Predictor	B	SE	*P*-value	BH-FDR *p*	95% CI
Intercept	10.4872	1.9905	<0.001	/	6.4540 to 14.5204
BFM	−0.0083	0.0238	0.729	0.948	−0.0567 to 0.0401
MM	0.0061	0.0412	0.884	0.956	−0.0778 to 0.0900
Sex	0.0449	0.5307	0.933	0.852	−1.0307 to 1.1206
Age	−0.1696	0.0647	0.013	0.048	−0.3006 to −0.0386
Model fit: R^2^ = 0.159, adjusted R^2^ = 0.068					

B, unstandardized regression coefficient; SE, standard error; CI, confidence interval; MM, muscle mass; BFM, body fat mass. BH-FDR *p*-values were calculated using the Benjamini–Hochberg false discovery rate correction for multiple testing. No evidence of problematic multicollinearity was observed, with all variance inflation factor (VIF) values below 5 (BFM = 1.13, MM = 2.87, sex = 3.30, age = 1.14). Model fit statistics were as follows: R^2^ = 0.159 and adjusted R^2^ = 0.068.

#### 3.6.3 Comparison of physical performance and body composition according to nutrition knowledge level

**Figure 3:**
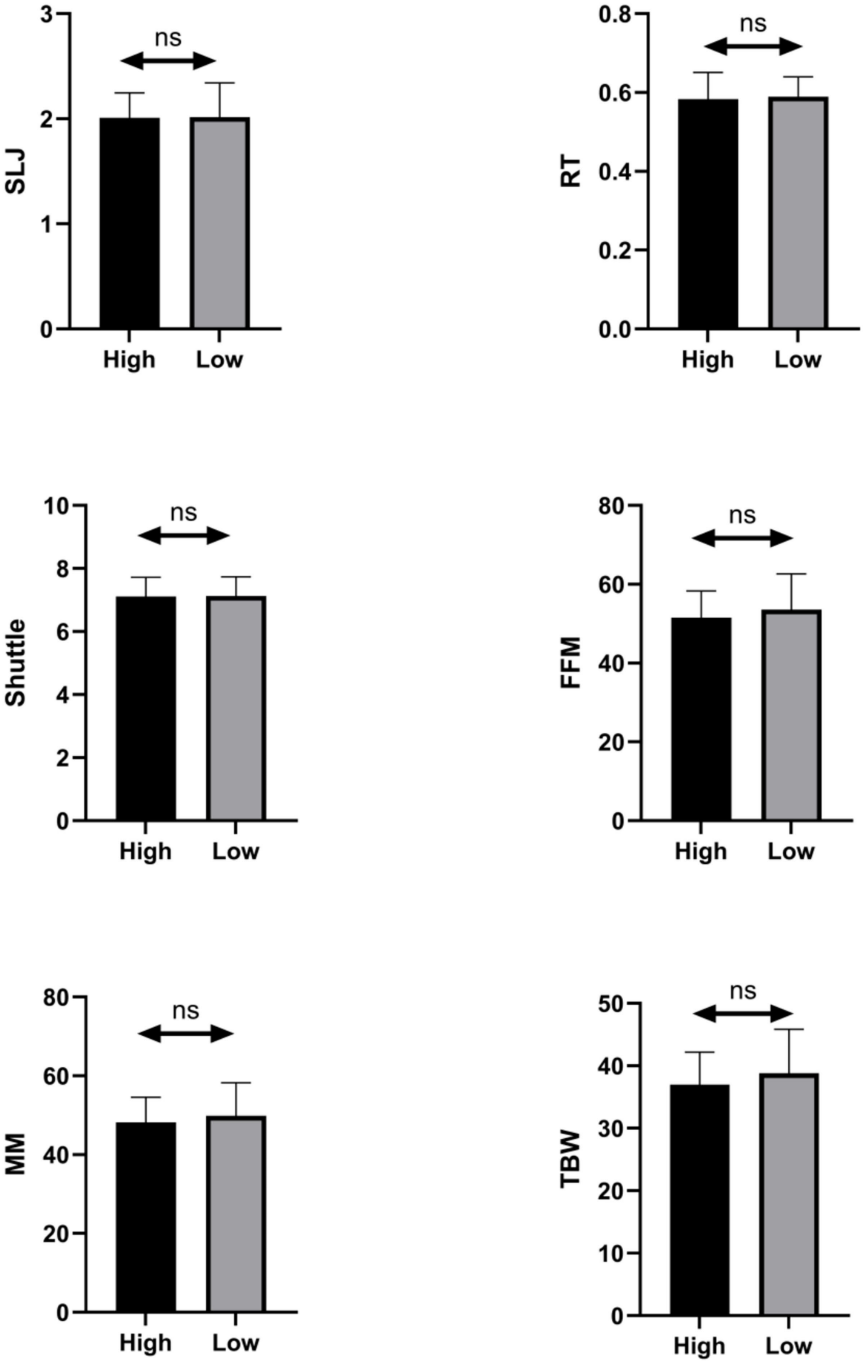
Comparison of physical performance and body composition between the relatively high-scoring group and the relatively low-scoring group. Data are presented as mean ± SD. Standing long jump (SLJ), reaction time (RT), shuttle run (Shuttle), fat-free mass (FFM), muscle mass (MM), and total body water (TBW), ns (*p >* 0.05).

After confirming normality using the Shapiro–Wilk test where applicable, independent-samples *t*-tests or Mann–Whitney U tests were performed to compare the High and Low nutrition-knowledge groups. As shown in Figure 3, no significant differences were observed between the two groups in standing long jump performance (SLJ), reaction time (RT), shuttle run performance, fat-free mass (FFM), muscle mass (MM), or total body water (TBW) (all *p >* 0.05). These findings indicate that nutrition knowledge was not significantly associated with these outcomes in the present sample. Still, they do not rule out meaningful associations in larger or more heterogeneous samples of athletes.

#### 3.6.4 Results of principal component analysis

**Figure 4:**
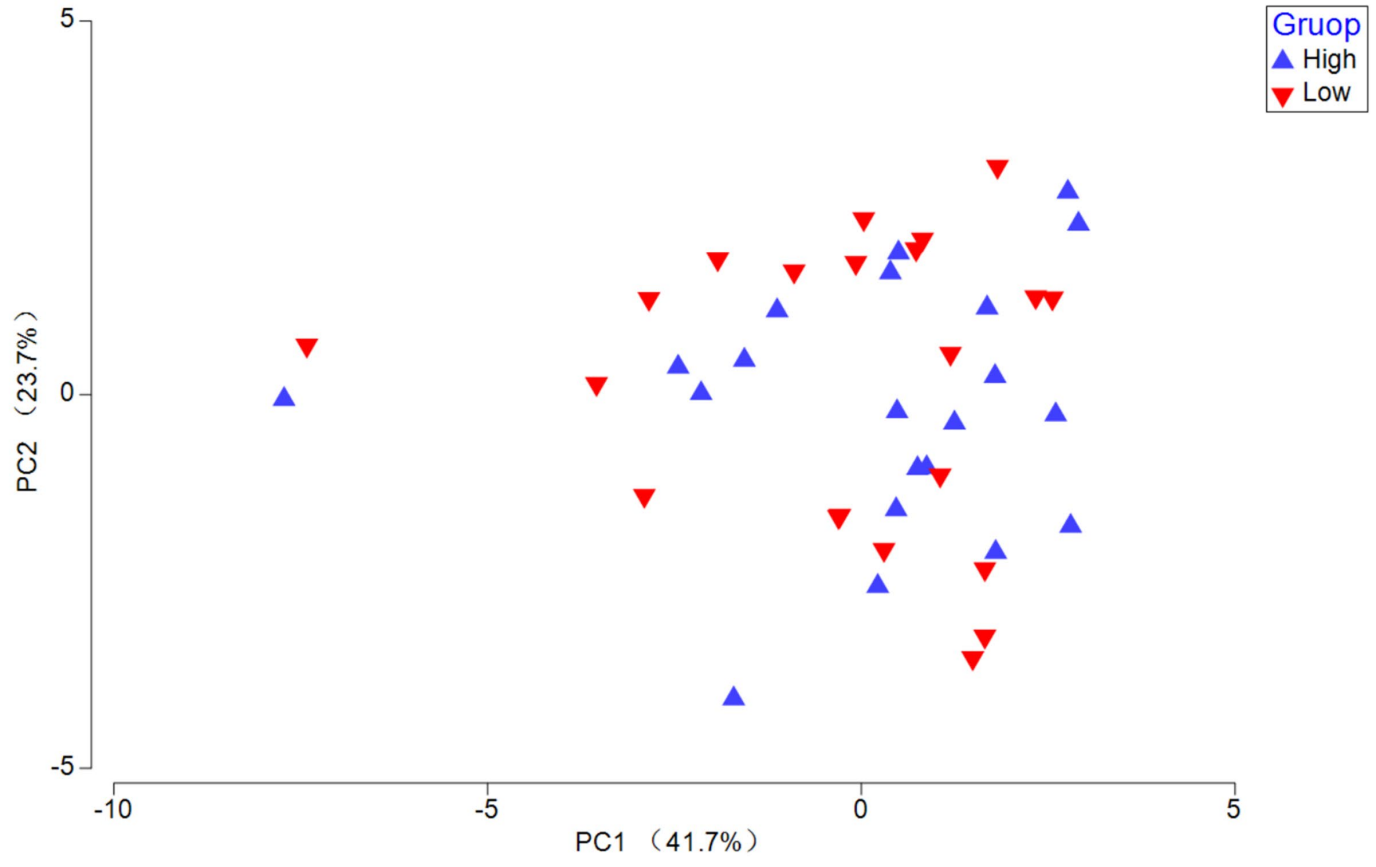
PCA of dietary intake and body composition variables. PC1 and PC2 summarize the main multivariate patterns among standardized dietary intake and body-composition variables. Blue upward triangles represent athletes with high nutrition knowledge, and red downward triangles represent athletes with low nutrition knowledge. Vector direction and length indicate the direction and relative contribution of variable loadings; overlapping group distributions indicate limited separation between nutrition-knowledge categories.

The overall KMO value was 0.732, indicating acceptable sampling adequacy, and Bartlett’s test of sphericity was statistically significant [*χ*^2^(91) = 734.03, *p <* 0.001], supporting the suitability of the correlation matrix for PCA. However, the individual measure of sampling adequacy for saturated fatty acid intake was below the conventional threshold of 0.50. Therefore, particularly given the small sample size, the PCA findings were interpreted as exploratory. PCA showed that the first two principal components explained 41.1 and 23.7% of the total variance, respectively (Figure 4). PC1 was characterized mainly by strong loadings for Fiber, Fe, Energy, Folate, and F&V, indicating that this axis primarily reflected overall dietary quality and nutrient intake patterns. PC2 was characterized mainly by strong loadings for MM, FFM, TBW, SFA, and Fat, suggesting a dimension related to lean body composition and fat-related intake. The PCA score plot showed partial overlap between high- and low-nutrition knowledge groups, with no clear separation, indicating that nutrition knowledge categories did not form distinct multivariate profiles. Given the small sample size, these PCA findings should be interpreted as an exploratory visualization of variable clustering rather than confirmatory evidence of group differences. In addition to the correlation and regression analyses, PCA provided an overall view of how correlated dietary and body-composition variables clustered within the sample. Its main contribution was descriptive, allowing the multivariate profiles of the two nutrition-knowledge groups to be visualized rather than providing further confirmatory evidence.

## 4 Discussion

### 4.1 Dietary intake patterns and nutritional inadequacies

This study demonstrated that although macronutrient distribution among table tennis athletes generally met recommended guidelines, overall dietary quality remained suboptimal, particularly in micronutrient intake and dietary diversity. This pattern has been widely reported in athletic populations, where adequate macronutrient intake often coexists with insufficient intake of micronutrients and fiber (22) (Gasazza et al., 2018).

A key observation was the low reported intake of dietary fiber, vitamin C, calcium, iron, and folate. These findings may be partly related to the low fruit and vegetable consumption reported in the present sample. Previous research has similarly indicated that some athletes consume fewer plant-based foods than recommended, which may be associated with lower micronutrient intake and antioxidant availability (26, 27). Such dietary patterns may be relevant to immune function, recovery, and oxidative-stress management (28); however, these outcomes were not directly assessed in the present cross-sectional study.

Another important observation was the relatively low reported energy intake. While this may partially reflect under-reporting inherent in the three non-consecutive 24-h recall method (two weekdays and one weekend day), it may also indicate inadequate energy availability (29, 30). Insufficient energy intake has been associated with reduced training adaptations, increased fatigue, and potential long-term health consequences (31). From a physiological perspective, energy availability represents a fundamental prerequisite for maintaining performance and supporting metabolic functions (32, 33). Overall, these findings suggest that athletes may prioritize macronutrient intake while overlooking dietary quality. This imbalance highlights the importance of shifting nutritional strategies from quantity-focused approaches toward nutrient density and food diversity.

### 4.2 Nutrition knowledge and its limited translation into practice

The results of the present study indicate that table tennis athletes possess an average level of nutrition knowledge, with relatively higher scores in basic domains and lower scores in sports-specific areas. This pattern is consistent with previous studies showing that athletes often demonstrate only average knowledge levels, particularly in applied sports nutrition contexts (22, 34).

Despite this, no significant differences were observed between the high- and low-nutrition knowledge groups in body composition or physical performance. This finding should be interpreted cautiously and does not indicate that nutrition knowledge is unimportant. Rather, within this sample, nutrition knowledge was not significantly associated with measurable differences in body composition or performance. Several explanations may account for this result, including a limited sample size, relatively homogeneous training backgrounds, questionnaire sensitivity, and barriers that prevent knowledge from translating into behavior. Actual dietary behavior is influenced by access to appropriate foods, time constraints, coaching environments, team culture, food availability, and individualized dietary support. Consistent with this interpretation, Alkasasbeh et al. (18) reported that social and demographic factors were associated with sports nutrition knowledge and food habits among university athletes. Therefore, nutrition education may need to be combined with practical behavior-change strategies and food-environment support.

### 4.3 Multidimensional relationships among diet, body composition, and performance

The correlation analyses indicated associations between indicators of lean body composition and explosive-power performance. This pattern is consistent with previous studies that have reported relationships among fat-free mass, muscle mass, and strength or power outcomes in athletes (35, 36). Dietary variables also exhibited clustering patterns, suggesting that overall dietary patterns may provide useful information beyond individual nutrient measures. For example, micronutrient variables tended to cluster together, while fiber intake was associated with fruit and vegetable consumption. However, these exploratory patterns should not be interpreted as evidence that dietary patterns determine nutritional status or performance.

After adjustment, the multiple regression analyses did not identify significant independent associations between the selected dietary or body composition variables and most performance outcomes. Moreover, the relatively low explanatory power of several models suggests that factors not assessed in this study may account for a substantial proportion of the variability in performance. Table tennis performance depends on neuromuscular coordination, reaction speed, technical skill, and tactical ability, which general physiological or nutritional measures may not adequately capture (1, 37). Similarly, the limited association between reaction time and nutritional variables suggests that cognitive and neural factors may be relevant. The observed association between age and shuttle-run performance may also reflect differences in training experience or adaptation, although this interpretation requires confirmation in larger studies (38). The use of general physical tests may also have contributed to the weak associations, as these tests do not capture the technical and perceptual demands of table tennis. In addition, the low adjusted R^2^ values for reaction time (0.060) and shuttle-run performance (0.068) indicate that these models explained little of the observed variation.

Overall, the relationships among dietary intake, body composition, and performance appear to be complex and multifactorial. Nutrition may be associated with performance through longer-term relationships with body composition, recovery, and physiological adaptation (39). Nevertheless, because of the cross-sectional design, small sample size, and predominantly non-significant regression findings, the present results cannot establish causal or mediating pathways and should be regarded as exploratory.

### 4.4 Limitations and practical implications

Several limitations should be considered when interpreting the present findings. The cross-sectional design precludes causal inference regarding the relationships among dietary intake, nutrition knowledge, body composition, and physical performance. Therefore, the observed relationships should be interpreted as associations rather than evidence that nutrition-related variables directly influence or determine performance. The relatively small sample size may also have reduced statistical power, particularly in the multivariable regression, PCA, and sex-stratified analyses. Some non-significant findings may consequently reflect type II error rather than the absence of meaningful associations. The small sex-specific subgroup sizes further limit the reliability and generalizability of the male–female comparisons. Accordingly, the findings should be regarded as preliminary and exploratory, and future multicenter studies should use larger samples and prospectively determined sample sizes. This concern is particularly relevant given the number of variables examined in only 42 participants. Thus, some non-significant results may reflect insufficient statistical power rather than a true lack of association.

Dietary intake was assessed using three non-consecutive 24-h recalls, including two weekdays and one weekend day. Although this approach provides more representative information than a single recall, it may not fully capture habitual intake and remains susceptible to recall bias, reporting errors, and dietary under-reporting (40). The low reported energy and micronutrient intakes may therefore reflect genuine dietary inadequacy, under-reporting, or both. Nevertheless, the limited consumption of fruits and vegetables provides some support for suboptimal dietary quality. Because objective energy expenditure was not measured, energy availability and the plausibility of the reported energy intake could not be determined. Future studies should combine multi-day dietary assessment with objective measures of energy expenditure or energy availability.

Body composition was assessed using bioelectrical impedance analysis. Although standardized pre-assessment procedures were implemented, BIA estimates may still be influenced by hydration status, recent food and fluid intake, glycogen fluctuations, recent training, and exercise-induced changes in body-fluid distribution. The physical performance tests also primarily assessed general change-of-direction ability, lower-limb explosive power, and reaction time. Although these capacities are relevant to table tennis, the tests did not directly assess sport-specific technical, perceptual, or tactical performance, including stroke accuracy, responses to table-tennis-specific stimuli, tactical decision-making, or match performance. This may limit the ecological validity of the findings. Future studies should consider more precise body-composition methods and combine general physical tests with validated table tennis-specific assessments.

The multivariate analyses should also be interpreted cautiously. Although the overall KMO value and Bartlett’s test supported the general suitability of the correlation matrix for PCA, the participant-to-variable ratio was relatively small. In addition, the individual measure of sampling adequacy for saturated fatty acid intake was 0.438, below the conventional threshold of 0.50. These factors may have reduced the stability and reproducibility of the component structure; therefore, the PCA findings should be considered an exploratory representation of multivariate patterns. This may have increased standard errors and reduced the stability of the individual regression coefficients.

Multiple statistical tests were conducted. To reduce the risk of false positives, the Benjamini-Hochberg false discovery rate correction was applied separately within the prespecified analysis families. Nevertheless, the large number of exploratory analyses and the small sample size still warrant cautious interpretation, particularly for isolated findings near the significance threshold. Furthermore, several potentially relevant factors were not assessed, including training load, sleep quality, psychological status, recovery strategies, habitual food availability, and access to professional nutrition support. Omission of these variables may have contributed to the limited explanatory power of the statistical models.

Despite these limitations, the study provides potentially useful practical information. The discrepancy between generally acceptable macronutrient distribution and inadequate intake of dietary fiber, micronutrients, and fruits and vegetables suggests that nutrition interventions should focus on dietary quality, nutrient density, and food diversity rather than macronutrient distribution alone. Nutrition education should also be combined with practical behavior-change strategies, structured meal planning, supportive food environments, and individualized guidance from qualified sports nutrition professionals.

## 5 Conclusion

This study provides an exploratory evaluation of dietary intake, nutrition knowledge, body composition, and physical performance among table tennis athletes. Although macronutrient distribution generally fell within recommended ranges, notable insufficiencies in dietary fiber, vitamin C, calcium, iron, folate, and fruit and vegetable intake were identified, suggesting suboptimal dietary quality. Nutrition knowledge among the athletes was average; however, it was not significantly associated with body composition or physical performance outcomes within this sample. Regression and PCA findings should be interpreted with caution because most associations were non-significant, the model’s explanatory power was limited, and the cross-sectional design precludes causal inference. These findings support the need for practical, individualized nutrition strategies and for future larger-scale studies that incorporate objective energy expenditure assessment, sex-specific analysis, and table tennis-specific performance indicators. These non-significant findings do not exclude possible relationships that a larger study might detect. They may also reflect reliance on general physical tests rather than table tennis-specific outcomes. The reaction-time and shuttle-run models explained little of the observed variation and should therefore not be given strong practical interpretation.

## Data Availability

The raw data supporting the conclusions of this article will be made available by the authors, without undue reservation.

## Footnotes

1
https://intake24.org/
